# MicroRNA‐351 eases insulin resistance and liver gluconeogenesis via the PI3K/AKT pathway by inhibiting FLOT2 in mice of gestational diabetes mellitus

**DOI:** 10.1111/jcmm.14079

**Published:** 2019-07-09

**Authors:** Shu‐Hong Chen, Xiao‐Nan Liu, Yan Peng

**Affiliations:** ^1^ Department of Endocrinology Linyi People's Hospital Linyi Shandong Province P.R. China

**Keywords:** FLOT2, gestational diabetes mellitus, insulin resistance, liver gluconeogenesis, microRNA‐351, PI3K/AKT pathway

## Abstract

Gestational diabetes mellitus (GDM) is known as different degree glucose intolerance that is initially identified during pregnancy. MicroRNAs (miRs) may be a potential candidate for treatment of GDM. Herein, we suggested that miR‐351 could be an inhibitor in the progression of GDM via the phosphoinositide 3‐kinase/protein kinase B (PI3K/AKT) pathway. Microarray analysis was used to identify differentially expressed genes and predict miRs regulating flotillin 2 (FLOT2). Target relationship between miR‐351 and FLOT2 was verified. Gestational diabetes mellitus mice were treated with a series of mimic, inhibitor and small interfering RNA to explore the effect of miR‐351 on insulin resistance (IR), cell apoptosis in pancreatic tissues and liver gluconeogenesis through evaluating GDM‐related biochemical indexes, as well as expression of miR‐351, FLOT2, PI3K/AKT pathway‐, IR‐ and liver gluconeogenesis‐related genes. MiR‐351 and FLOT2 were reported to be involved in GDM. FLOT2 was the target gene of miR‐351. Gestational diabetes mellitus mice exhibited IR and liver gluconeogenesis, up‐regulated FLOT2, activated PI3K/AKT pathway and down‐regulated miR‐351 in liver tissues. Additionally, miR‐351 overexpression and FLOT2 silencing decreased the levels of FLOT2, phosphoenolpyruvate carboxykinase, glucose‐6‐phosphatase, fasting blood glucose, fasting insulin, total cholesterol, triglyceride, glyeosylated haemoglobin and homeostasis model of assessment for IR index (HOMA‐IR), extent of PI3K and AKT phosphorylation, yet increased the levels of HOMA for islet β‐cell function, HOMA for insulin sensitivity index and glucose transporter 2 expression, indicating reduced cell apoptosis in pancreatic tissues and alleviated IR and liver gluconeogenesis. Our results reveal that up‐regulation of miR‐351 protects against IR and liver gluconeogenesis by repressing the PI3K/AKT pathway through regulating FLOT2 in GDM mice, which identifies miR‐351 as a potential therapeutic target for the clinical management of GDM.

## 1 INTRODUCTION

In Asia, it is reported that the incidence of diabetes is rapidly increasing and that no less than 60% of the patients diagnosed with diabetes is alive.^1^ Diabetes is one of the most common diseases among Chinese population which has been specially analysed in Chinese reports.^2^, ^3^ Gestational diabetes mellitus (GDM) is a cumulatively obstetric disorder and a complication with variable severity of glucose intolerance when pregnancy is initially recognized.^4^ Insulin resistance (IR) is associated with GDM, and gluconeogenesis is also involved in GDM due to correlation with IR.^5^, ^6^ Multiple gestation is a factor associated with rising risk of GDM.^7^ Varying other factors, including genetic factors and environmental ones, may impact the pathogenesis of GDM, and a prevalent theory is that GDM is arisen from IR in pregnancy and the metabolic defects of β‐cell dysfunction.^8^ Both the mother and her children may be negatively affected by both short‐ and long‐term adverse health outcome brought by GDM.^3^ Nowadays, women still desire for intensive counselling and treatment for GDM around the world,^9^ so it is urgent to elucidate the molecular mechanisms underlying GDM development and identify novel prognostic markers and molecular therapeutic targets for improving the diagnosis, prevention and treatment of GDM.

Flotillins are known to be correlated with vesicular invaginations of the plasma membrane and regulation of signal transduction.^10^ Recently, flotillin 2 (FLOT2) was identified to be a newly discovered target for miR‐34a in melanoma.^11^ Evidence showed that FLOT2 was in interaction with signalling molecules such as receptors, kinases, adhesion molecules and G proteins in a direct manner.^12^ In addition, a previous study revealed that FLOT2 was an insulin pathway in type 2 diabetes mellitus (T2DM).^13^ Furthermore, GSE87295 database revealed that FLOT2 was up‐regulated in GDM patients compared with the normal individuals. The association between FLOT2 and phosphoinositide 3‐kinase/protein kinase B (PI3K/AKT3) pathway has been explored and the mechanism was dependent on MMPs expression, E‐cadherin expression, Foxo1 activity and cell cycle arrest.^14^ Besides, a earlier finding indicated the role of PI3K/AKT pathway in diabetes.^15^ The participation of PI3K/AKT pathway in GDM was pointed out in a previous paper and sex hormone‐binding globulin exerts a function during the process by affecting the insulin signal transduction pathway.^16^ MicroRNAs (miRs) are recognized as small endogenous RNAs which account for altering gene‐expression post‐63 transcriptionally and different biological process.^17^, ^18^ Furthermore, miR‐351 was presumed to bind to the 3'‐untranslated region (3'‐UTR) of FLOT2 in microRNA.org. MicroRNA‐351 is a family member of the interferon β (INFβ)‐inducible mRNAs which plays a role in accelerating cellular antiviral activities.^19^ MicroRNA‐351 level is temporarily elevated in the period of muscle regeneration and implicated in muscle atrophy.^20^ Based on the aforementioned literature, we could suggest that miR‐351 regulated GDM by mediating FLOT‐dependent PI3K/AKT pathway. In this current study, we aimed to identify the role of miR‐351 in GDM by down‐regulating FLOT via PI3K/AKT pathway.

## 2 MATERIALS AND METHODS

### 2.1 Ethic statement

This animal experiment was approved by the animal ethics committee of Linyi People's Hospital. In addition, best efforts were made to minimize the suffering of animals.

### 2.2 Microarray analysis

The GDM related gene expression chip GSE87295 was downloaded from the Gene Expression Omnibus (GEO) database (https://www.ncbi.nlm.nih.gov/geo/), which included vascular endothelial cell gene expression data of five GDM samples and five control samples. The annotated platform for GSE87295 was the GPL10558‐Illumina HumanHT‐12 V4.0 expression beadchip. The differential analysis was carried out with R. Software "limma" package^21^ after obtaining chip expression matrix and annotation information. The threshold values were set as *P* < 0.05 and $|\mathrm{logFoldChange}\left(\mathrm{FC}\right)|>2$. And then thermogram map of differentially expressed genes was plotted. In the DisGeNET database (http://www.disgenet.org/web/DisGeNET/menu/search?4), “Gestational Diabetes” was set as the key word to screen GDM‐related genes. And then the interaction between GDM differentially expressed genes and disease‐related genes was analysed by the String database (https://string-db.org/). The interaction network was visualized by the Cytoscape 3.6.0 software.^22^ Further prediction of targeting miR of differentially expressed genes was conducted by miRNAMap (http://34.236.212.39/microrna/getGeneForm.Do), miRWalk (http://mirwalk.umm.uni-heidelberg.de/), miRDB (http://www.mirdb.org/), DIANA (http://diana.imis.athena-innovation.gr/DianaTools/index.php?r = microT_CDS/index) and TargetScan (http://www.targetscan.org/vert_71/). Finally, the jvenn tool (http://jvenn.toulouse.inra.fr/app/example.html) was applied for comparison of miR prediction results.

### 2.3 Dual luciferase reporter gene assay

Biological site https://cm.jefferson.edu/rna22/Interactive was used to predict and analyse the target gene of miR‐351 and identify if FLOT2 was a direct target gene of miR‐351. Further dual‐luciferase reporter gene assay was employed to confirm the targeting relationship between FLOT2 and miR‐351. Fragments of synthetic FLOT2 3'UTR were introduced into the pMIR‐reporter using the endonuclease site SpeI and Hind III. The complementary sequence mutation sites of seed sequences were designed on the FLOT2‐wild type (Wt), and the target fragment was inserted into the reporter plasmid of pMIR‐reporter using T4 DNA ligase after restriction endonuclease digestion. The correctly sequenced FLOT2‐Wt and FLOT2‐mutant type (Mut) were cotransfected with miR‐351 in a respective manner into the HEK‐293T cells (CRL‐1415, Shanghai Xin Yu Biotech Co., Ltd., Shanghai, China). After transfection of 48 hours, cells were collected, lysed and centrifuged for 3‐5 minutes with the supernatants collected. The luciferase assay reagent and Renilla luciferase assay buffer were dissolved by using a luciferase assay kit (RG005, Beyotime Biotechnology Co., Shanghai, China), respectively. One hundred‐microlitre sample buffer was added with substrate at a ratio of 1:100 to prepare Renilla luciferase assay working solution, and the fluorometer was operated. A total of 20‐100 μL sample from each group was mixed with 100 μL of luciferase assay buffer, percussed evenly with pipette to determine relative luminescence units (RLU). With Renilla luciferase as reference, luciferase activity was RLU of firefly luciferase/RLU of Renilla luciferase.^23^

### 2.4 Experimental animals

Ninety C57BL/6J female mice of clean grade (weighing 23 ± 2 g; aging 8 ± 2 weeks) were purchased from Beijing Vital River Laboratory Animal Technology Co., Ltd. (Beijing, China, SCXK (Jing) 2012‐0001). Mice were housed under the temperature of about 25°C with a relative humidity of 38%‐68% and natural lighting. Mice were fed with standard food with free access to water and food. The experiment was conducted after adaptive feeding for 1 week.

### 2.5 Establishment of GDM mouse model

The GDM mouse model was established by intraperitoneal injection of streptozotocin (STZ). Before the experiment, venous blood of mouse tail was collected to determine the fasting blood glucose (FBG), with 3‐5 mmol/L as the normal blood glucose value. The vaginal smear method was used to determine the oestrus cycle of mice. The female and male mice in the early stage of oestrus were mated. The vaginal opening and litter were checked on the next morning to observe whether there was vaginal suppository in mice. The observed vaginal suppository in mice was regarded as the 1st day of pregnancy. Eighty‐two of the 1st of pregnancy mice were fasted on the morning of the 6th day of pregnancy with free access to water for 12 hours. A total of 10 mice were randomly selected as normal group. The other 72 mice were intraperitoneally injected with 40 mg/kg of STZ (STZ was dissolved in 0.1 mol/L citric acid/sodium citrate buffer [pH = 4.2] and preserved in ice bath). Mice in the normal group were intraperitoneally injected with equal amount of citric acid/sodium citrate buffer. On the 7th day after injection, FBG ≥ 11.1 mmol/L was regarded as the successful establishment of GDM mouse model.^24^ Finally, 60 mice were successfully modelled, and randomly assigned to six groups with 10 mice for each group. The experiments had seven groups (10 mice in each group): normal group (normal pregnancy mice), GDM group (GDM mice), negative control (NC) group (GDM mice injected with scramble small interfering RNA [siRNA]), miR‐351 mimic group (GDM mice injected with miR‐351 mimic), miR‐351 inhibitor group (GDM mice injected with miR‐351 inhibitor), siRNA‐FLOT2 group (GDM mice injected with siRNA‐FLOT2) and miR‐351 inhibitor + siRNA‐FLOT2 group (GDM mice injected with miR‐351 inhibitor and siRNA‐FLOT2). Scramble siRNA, miR‐351 mimic, miR‐351 inhibitor (10 μg of miR‐351 inhibitor and 10 μg of si‐FLOT2 in the miR‐351 inhibitor + siRNA‐FLOT2 group) with 20 μg of each were dissolved in 2.5 mL of saline, respectively, and then rapidly injected into the tail vein of mice, and mice in the normal and GDM groups were injected with the equal amount of saline.

### 2.6 Haematoxylin‐eosin staining

After 6 weeks of injection, mice of each group were randomly selected for pancreatic tissues extraction. The tissues were fixed in 4% paraformaldehyde, decalcified, paraffin‐embedded and stored at 4°C. After tissues were sliced into 5 μm sections, haematoxylin‐eosin staining was performed. First, sections were stained with haematoxylin for 5‐10 minutes, immersed in 70% ethanol for 30 minutes to remove cytoplasm colouring, alkalized with alkaline solution and washed with distilled water for 1 minute. Second, sections were stained with eosin for 30‐60 seconds, dehydrated with gradient ethanol, cleared two times with xylene, dried and mounted. Finally, the morphological structures of the pancreatic tissues were observed under an optical microscope. The nucleus was generally stained into black‐blue, and the cytoplasm was pale red. The apoptotic cells were scattered in the pancreatic tissues, and the necrotic tissues were stained into red without structure and the stained nuclear disappeared.

### 2.7 Terminal deoxynucleotidyl transferase‐mediated dUTP nick‐end labeling assay

After 6‐week administration, pancreatic tissues from mice in each group were resected, collected and stained with cell apoptotic enzyme streptavidin peroxidase using terminal deoxynucleotidyl transferase‐mediated dUTP nick‐end labeling (TUNEL) assay in accordance with the instructions of cell apoptosis in situ detection kit. Then, the tissues were observed under an optical microscope, with experimental data and results recorded in detail. Afterwards, five high power fields with the most positive cell number (brown‐stained nucleus) (×400) were selected from TUNEL positive sections in each group. The percentage of positive cells in 500 cells was calculated as apoptotic index.^25^

### 2.8 Periodic acid‐Schiff staining

After 6 weeks of injection, mice of each group were randomly selected for liver tissue extraction. The tissues were fixed in 4% paraformaldehyde, decalcified, paraffin‐embedded, stored at 4°C and sliced into 5 μm sections. The sections were dewaxed, stained with 0.5% periodic acid solution for 10 minutes and rinsed with distilled water for 10 minutes. Afterwards, the sections were stained with 100 μL of Schiff solution for 15 minutes, and 100 μL of 0.5% sodium bisulfite solution was added for 2 minutes reaction after the removal of Schiff solution. Finally, sections were rinsed under running water for 10 minutes, stained with haematoxylin for 1 minute, washed with water, dried and mounted with neutral gel. The morphological structures of liver tissue were observed under an optical microscope.

### 2.9 Detection of biochemical indexes

After 6 weeks of injection, venous blood of mice tail was extracted from each group 1 day before killing, and centrifuged at 1811× g for 15 minutes with the serum collected. Glucose oxidase method was used to measure the content of FBG. The glucose kit was provided by Beijing Zhongsheng Beikong Biochemistry Company (Beijing, China), and the instrument was automatic biochemical analyzer (7171A). The level of fasting insulin (FINS) in serum was measured by mouse insulin ELISA kit (Shanghai Bluegene Biotech Co., Ltd., Beijing, China). The total cholesterol (TC) and triglyceride (TG) were assessed by the end‐point method using the automatic biochemical analyzer (7171A). Based on the measured content of FBG and FINS, the homeostasis model of assessment (HOMA) for IR index (HOMA‐IRI), HOMA for islet β‐cell function (HOMA‐β%) and HOMA for insulin sensitivity index (HOMA‐ISI) were calculated and compared. $HOMA-IRI=\mathrm{FBG}\times \frac{\mathrm{FINS}}{22.5}$; $HOMA-ISI=\ln\left[\frac{1}{\left(\mathrm{FBG}\times \mathrm{FINS}\right)}\right]$; $HOMA-\beta \%=20\times \frac{\mathrm{FINS}}{\left(\mathrm{FBG}-3.5\right)}$.

### 2.10 Reverse transcription quantitative polymerase chain reaction (RT‐qPCR)

The liver tissues of mice in each group were grounded into uniform fine powder. The total RNA was extracted using Trizol (Invitrogen Inc., Carlsbad, CA, USA). The ratio of optical density (OD) _260 nm_/OD_280 nm_ was measured using an ultraviolet spectrophotometer to determine the purity of the RNA and to ensure that the OD value was between 1.8 and 2.1. The miR was isolated using the PureLink FFPE Total RNA Isolation Kit (Shanghai Haoran Biotech Co., Ltd., Shanghai, China). MiR‐351 and RNAs of other samples were reversely transcribed into cDNA using the ALL‐in‐one miRNA reverse transcription kit (GeneCopoeia, Rockville, MD, USA). The reactions were performed in a total volume of 25 μL, including 2 μL template RNA, 1 μL PolyA Polymerase, 1 μL RTase Mix, 5 μL of 5× Reaction Buffer and 16 μL distilled water. After being mixed well, the mixture was placed in water bath at 37°C for 60 minutes and 85°C for 5 minutes. The obtained cDNA was temporarily stored in a refrigerator at −80°C for subsequent PCR reaction. Repeated freezing and thawing should be avoided. The fluorescence quantitative PCR was performed according to the instructions of SYBR^®^ Premix Ex TaqTM II Kit (TaKaRa, Dalian, Liaoning, China). Based on mRNA PCR reaction system, 2 μL first chain product, 0.8 μL forward, 0.8 μL reverse primer and 10.4 μL of Ultra SYBR Mixture were mixed together with 2 μL of cDNA template, and the reaction system was brought up to 20 μL with distilled water. The reaction system was mixed evenly, centrifuged to remove bubbles and PCR reaction was performed on an ABI 7500 PCR instrument. The reaction conditions consisted of pre‐denaturation at 95°C for 30 seconds, and 40 cycles of denaturation at 95°C for 5 seconds, anneal at 60°C for 34 seconds and extension at 72°C for 1 minute, finally extension at 72°C for 7 minutes. U6 was used as an internal reference of miR‐351 and β‐actin for other genes. The primers of U6, β‐actin, miR‐351, FLOT2, PI3K, AKT, phosphoenolpyruvate carboxykinase (PEPCK), glucose‐6‐phosphatase (G‐6‐Pase) and glucose transporter 2 (GLUT2) were designed and synthesized by Wuhan Bio Just Bioengineering Co., Ltd. (Wuhan, Hubei, China) (Table 1). The 2^−ΔΔCt ^represents the ratio of the target gene expression between the GDM group and the control group: $\Delta \Delta \mathrm{CT}=\Delta {\mathrm{Ct}}_{\mathrm{experimental}\;\mathrm{group}}-\Delta {\mathrm{Ct}}_{\mathrm{control}\;\mathrm{group}}$, $\Delta \mathrm{Ct}={\mathrm{Ct}}_{\mathrm{target}\;\mathrm{genes}}-{\mathrm{Ct}}_{\frac{U6}{\beta -actin}}$. Ct referred to the amplification cycles when the real‐time fluorescence intensity reached the set threshold value and the amplification entered a logarithmic growth. The experiment was repeated three independent times, and miR‐351 expression and mRNA levels of FLOT2, PI3K, AKT, PEPCK, G‐6‐Pase and GLUT2 in each group after transfection were determined.

**Table 1:** Primer sequences for RT‐qPCR

Gene	Forward (5ʹ‐3ʹ)	Reverse (5ʹ‐3ʹ)
miR‐351	AGCCCTTTGAGCCTGGAGTG	TTTAACACTCTTCTCCAGTTCCCAG
FLOT2	CGCTGTGAGGACGTAGAGAC	GCAGCACGACGTTCTTAATGT
PI3K	ACACCACGGTTTGGACTATGG	GGCTACAGTAGTGGGCTTGG
AKT	ATGAACGACGTAGCCATTGTG	TTGTAGCCAATAAAGGTGCCAT
PEPCK	GACCATAACATAGTATACACCTGCTGC	AGAAGGGTCGCATGGCAA
G‐6‐Pase	CACCTGTGAGACCGGACCA	GACCATAACATAGTATACAC‐CTGCTGC
GLUT2	TACGGCAATGGCTTTATC	CCTCCTGCAACTTCTCAAT
U6	GCTTCGGCAGCACATATACTAAAAT	CGCTTCACGAATTTGCGTGTCAT
β‐actin	GTGACGTTGACATCCGTAAAGA	GCCGGACTCATCGTACTCC

Notes

AKT, protein kinase B; FLOT2, flotillin 2; G‐6‐Pase, glucose‐6‐phosphatase; GLUT2, glucose transporter 2; miR‐351, microRNA‐351; PI3K, phosphoinositide 3‐kinase; PEPCK, phosphoenolpyruvate carboxykinase; RT‐qPCR, reverse transcription quantitative polymerase chain reaction.

### 2.11 Western blot analysis

The total protein was extracted from grinding liver tissues in each group using radio‐immunoprecipitation assay (RIPA) kit (R0010, Beijing Solarbio science & technology Co., Ltd., Beijing, China). The transfected cells were washed three times with pre‐cooled phosphate buffer saline. Each cell flask was added with an appropriate amount of protein lysate [60% RIPA + 39% sodium dodecyl sulphate (SDS) + 1% protease inhibitor]. Cells were collected into an eppendorf tube, and lysed on ice for 30 minutes. Then cells were centrifuged at 4°C at 25764× g for 30 minutes, and the supernatant was collected and placed in ice box which was further used for determination of protein concentration using bicinchoninic acid kit (PC0020, Beijing Solarbio science & technology Co., Ltd.). Next, 10% separation gel and 5% concentrated gel were prepared by using SDS‐PAGE kit. Proteins were separated by electrophoresis on polyacrylamide gel, and subsequently transferred onto a polyvinylidene fluoride (PVDF) membrane by wet method. The PVDF membrane was blocked in 5% bovine serum albumin for 1 hour at room temperature, incubated for 30 minutes at room temperature with the addition of following diluted primary antibodies: rabbit anti‐mouse polyclonal antibody PI3K (1:1000, ab151549), rabbit polyclonal antibody p‐PI3K (1:1000, ab182651), rabbit polyclonal antibody AKT (1:1000, ab64148), p‐AKT (1:500, ab8933), rabbit polyclonal antibody FLOT2 (1:1000, ab154110), rabbit polyclonal antibody PEPCK (1:1000, ab187145), rabbit polyclonal antibody G‐6‐Pase (1:250, ab96142), rabbit polyclonal antibody GLUT2 (1:1000, ab95256) and β‐actin (1:1000, ab5694). All the above antibodies were purchased from Abcam (Cambridge, MA, USA). Next, the membrane was incubated with horseradish peroxidase‐labelled immunoglobulin G (1:5000, A21020, AmyJet Scientific Inc., Wuhan, Hubei, China) for 1 hour at room temperature, added with Tris‐buffered saline with Tween 20, oscillated and finally washed three times with 10 minutes for each time. Equal amounts of A solution and B solution in enhanced chemiluminescence detection kit (BB‐3501, Amersham LifeScience, Buckinghamshire, UK) were mixed evenly under conditions void of light, and then added onto the PVDF membrane. The images were captured by using a gel imager, and the membrane was photographed by the Bio‐Rad Image Analysis System (Bio‐Rad, Inc., Hercules, CA, USA), and analysed by Quantity One v4.6.2 software (Bio‐Rad, Inc.). Finally, the ratio of the grey value of the target protein bands/β‐actin protein bands was expressed as the relative protein level. The experiment was repeated three independent times.

### 2.12 Statistical analysis

spss 21.0 (IBM Corp., Armonk, NY, USA) was employed for statistical analysis. The experiment was repeated three independent times. The measurement data were displayed as mean ± SD. All data were tested for normal distribution and homogeneity of variance. Comparison between two groups was performed with *t* test, and comparisons among multiple groups with one‐way anova. *P < *0.05 was considered to be statistically significant.

## 3 RESULTS

### 3.1 MiR‐351 mediates the PI3K/AKT pathway by regulating FLOT2 in GDM

**Figure 1:**
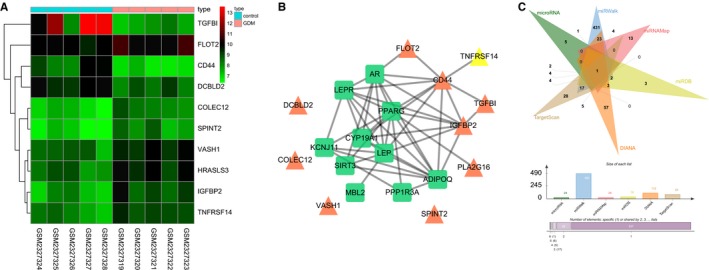
MiR‐351 plays a role in GDM through regulation of FLOT2 and the PI3K/AKT pathway. (A) the thermal map of the top 10 differentially expressed genes screened out from the GSE87295 chip. The abscissa indicated the sample number, the ordinate indicated the differentially expressed genes, and the upper right histogram was the colour gradation. Each rectangle corresponded to value of one sample expression with red stands for high‐expression and blue for low‐expression; (B) interaction network between differentially expressed genes and GDM‐related genes. Green squares represented GDM‐related genes, and orange triangles represented differentially expressed genes; (C) prediction results of FLOT2 targeting miR in miRNAMap, miRWalk, microRNA, miRDB, DIANA and TargetScan. MiR‐351, microRNA‐351; FLOT2, flotillin 2; PI3K, phosphoinositide 3‐kinase; AKT, protein kinase B; GDM, gestational diabetes mellitus; IGFBP2, insulin‐like growth factor‐binding protein 2

Initially, 106 differentially expressed genes were screened out from the GSE87295 chip using limma package of R language under the condition of *P* < 0.05 and |logFC|>2. The thermal map of top 10 differentially expressed genes was plotted by increment order according to *P* value for following analysis (Figure 1A). Gestational diabetes mellitus‐related genes were retrieved in the DisGeNET with “Gestational Diabetes” set as the key word, and the top 10 GDM‐related genes were selected as disease‐related genes. The interaction between top 10 differentially expressed genes and disease‐related genes was analysed using the String database, and the interaction network was plotted (Figure 1B). The differentially expressed genes that interacted with disease‐related genes in the interaction network were FLOT2, CD44 and IGFBP2, which revealed that these three genes may affect GDM. There were studies showing that CD44^26^ and IGFBP2^27^, ^28^ were related to GDM. Therefore, we mainly focused on the role of FLOT2 in GDM. As shown in Figure 1A, the expression of FLOT2 was higher in GMD samples than that in control samples. Moreover, it was demonstrated that the alteration of the PI3K/AKT pathway could influence GDM,^29^ and other studies revealed that FLOT2 could regulate the PI3K/AKT pathway.^14^, ^30^ With this respect, we speculated that the differential expression of FLOT2 may regulate the PI3K/AKT pathway in GDM. The target miRs of FLOT2 were predicted using miRNAMap, miRWalk, miR, miRDB, DIANA and TargetScan. Twenty‐four and 38 miRs were screened out from miR and miRDB, respectively. Twenty‐six miRs were screened out from miRNAMap with score >150, 490 miRs from miRWalk with energy <−25, 112 miRs from DIANA with miTG score >0.7 and 84 miRs from TargetScan with context++ score <−0.2. Venn map was drawn after comparisons of the above screened miRs (Figure 1C), and it turned out that only mmu‐miR‐351‐5p was in the intersection with potential possibility to regulate FLOT2. According to the results of bioinformatics analysis, a conclusion could be drawn that miR‐351 might play a role in regulation of the PI3K/AKT pathway to affect the expression of FLOT2 in GDM.

### 3.2 FLOT2 is a target gene of miR‐351

**Figure 2:**
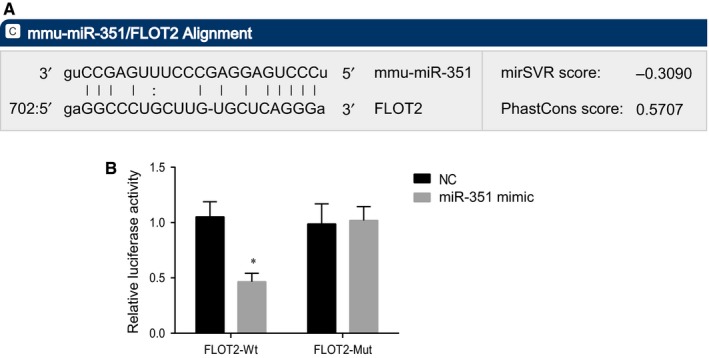
MiR‐351 directly targets FLOT2. (A) binding sequences of miR‐351 and FLOT2 3ʹUTR; (B) the luciferase activity of 3ʹ‐UTR in FLOT2‐Wt and FLOT2‐Mut; *, *P* < 0.05 vs the NC group; miR‐351, microRNA‐351; FLOT2, flotillin 2; Wt, wild type; Mut, mutant; UTR, untranslated region; the results were analysed by the paired *t* test, and the experiment was repeated three times

The targeting relationship between miR‐351 and FLOT2 was predicted by bioinformatic website (https://cm.jefferson.edu/rna22/Interactive). There was a specific binding region between the sequences of miR‐351 and FLOT2 (Figure 2A). Further, dual luciferase report gene assay was performed to verify that miR‐351 could bind to FLOT2. As shown in Figure 2B, compared with the NC group, the luciferase activity of 3’UTR in FLOT2‐Wt was significantly inhibited by miR‐351 mimic (*P* < 0.05), while the luciferase activity of 3ʹUTR in FLOT2‐Mut showed no significant difference (*P* > 0.05). These findings demonstrated that miR‐351 could specifically bind to FLOT2‐3ʹUTR, and down‐regulated the expression of FLOT2 after transcription.

### 3.3 miR‐351 overexpression and FLOT2 silencing reduce pancreatic cell apoptosis and alleviate GDM in mice

**Figure 3:**
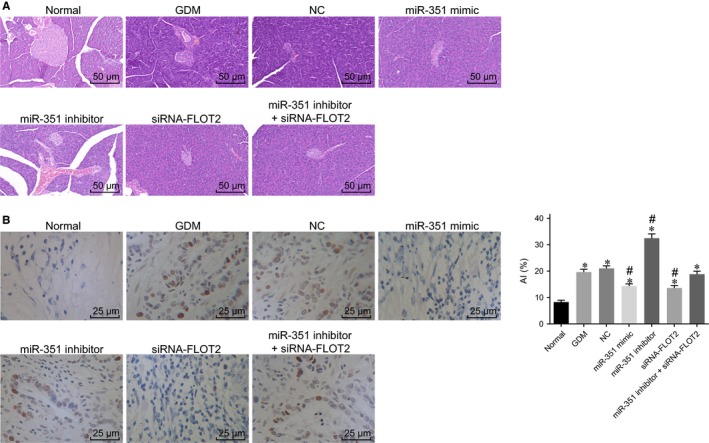
Overexpression of miR‐351 and silencing of FLOT2 promote regeneration of pancreatic islet and inhibit pancreatic cell apoptosis. (A) Haematoxylin and eosin staining result of pancreatic tissues in each group (×200); (B) cell apoptosis in mice pancreatic tissues detected by TUNEL assay (×400); *, *P* < 0.05 vs the normal group; #, *P* < 0.05 vs the GDM and NC groups; &, *P* < 0.05 vs the siRNA‐FLOT2 group; the results were analysed by the one‐way anova, and the experiment was repeated three times. MiR‐351, microRNA‐351; FLOT2, flotillin 2; NC, negative control; HE, haematoxylin‐eosin; siRNA, small interfering RNA; TUNEL, Terminal deoxynucleotidyl transferase‐mediated dUTP nick‐end labeling

Haematoxylin‐eosin staining was performed to observe the pathological changes of the pancreas under different transfection. As shown in Figure 3A, the endocrine and exocrine parts of the pancreas could be seen under the optical microscope. The exocrine part was serous acini with dark staining, and the endocrine part was scattered islets with shallow staining. The pancreatic islets of mice in the normal group were round or oval cell clusters with obvious boundaries and no membranes. A large number of islets and cells in the pancreatic islets were observed. Compared with the normal group, the atrophy of the pancreatic islets in the other six groups was increased with decreased cells in the pancreatic islets, aggravated inflammatory lesions and increased vacuolar degeneration of β cells. In contrast to the GDM and NC groups, the miR‐351 mimic, siRNA‐FLOT2 and miR‐351 inhibitor + siRNA‐FLOT2 groups had restored the structure of pancreatic islets with increased number of islets, increased number of cells in the islet, alleviated inflammatory lesions and promoted regeneration of pancreatic islet. While an opposite trend was found in the miR‐351 inhibitor group. The miR‐351 inhibitor + siRNA‐FLOT2 group exhibited increased atrophy of the pancreatic islets with decreased cells in the pancreatic islets, aggravated inflammatory lesions and increased vacuolar degeneration of β cells in comparison with the siRNA‐FLOT2 group. Planned comparisons revealed that overexpression of miR‐351 and silencing of FLOT2 could contribute to regeneration of pancreatic islets, thus alleviating GDM in mice.

Next, TUNEL assay was conducted to measure cell apoptosis in pancreatic tissues. Results (Figure 3B) showed that compared with the normal group, other six groups had obviously increased cell apoptosis in pancreatic tissues (all *P* < 0.05). In comparison with the GDM and NC groups, cell apoptosis in pancreatic tissues was significantly decreased in the miR‐351 mimic, siRNA‐FLOT2 and miR‐351 inhibitor + siRNA‐FLOT2 groups (all *P* < 0.05), while markedly increased in the miR‐351 inhibitor group (*P* < 0.05). When compared with the siRNA‐FLOT2 group, the miR‐351 inhibitor + siRNA‐FLOT2 group exhibited prominently elevated cell apoptosis in pancreatic tissues (all *P* < 0.05). Hence, miR‐351 overexpression and FLOT2 silencing inhibited cell apoptosis in pancreatic tissues.

### 3.4 miR‐351 elevation and FLOT2 depletion improve pathological changes of liver cells in GDM mice

**Figure 4:**
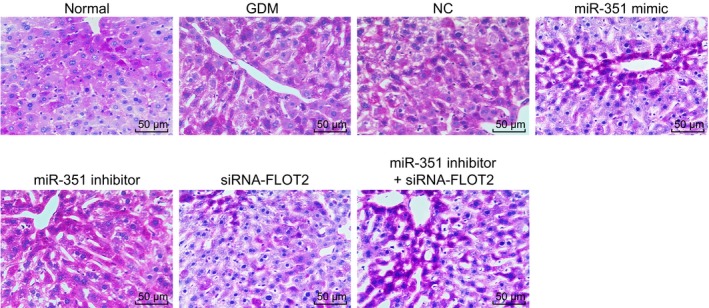
Overexpression of miR‐351 and silencing of FLOT2 decrease glycogen granules and nucleus vacuoles in liver cells of GDM mice (×200). MiR‐351, microRNA‐351; FLOT2, flotillin 2; GDM, gestational diabetes mellitus; NC, negative control; PAS, periodic acid‐Schiff; siRNA, small interfering RNA

Periodic acid‐Schiff (PAS) staining was performed to observe the pathological changes of liver cells of GDM mice under different transfection. As shown in Figure 4, the normal group had a dense and well‐organized cell structure with uniform PBA staining. In contrast to the normal group, the other six groups had distorted and disorganized cell morphology, increased intracellular and intercellular PAS staining with obvious glycogen granules (stained into purple‐red) and nucleus vacuoles. Compared with the GDM and NC groups, the miR‐351 mimic, siRNA‐FLOT2 and miR‐351 inhibitor + siRNA‐FLOT2 groups exhibited alleviated morphological deformation, ordered arrangement and reduced intracellular and intercellular PAS staining with obviously decreased glycogen granules and nucleus vacuoles. While an opposite trend was found in the miR‐351 inhibitor group. The miR‐351 inhibitor + siRNA‐FLOT2 group showed aggravated morphological deformation, and increased intracellular and intercellular PAS staining with obviously increased glycogen granules and nucleus vacuoles in comparison to the siRNA‐FLOT2 group.^31^ All above, it was cleared that the pathological changes of liver cells could be alleviated by overexpressing miR‐351 and silencing of FLOT2 in GDM mice.

### 3.5 Overexpression of miR‐351 and silencing of FLOT2 promote insulin sensitivity and rescue islet β‐cell function in GDM mice

Glucose oxidase method was used to measure the content of FBG, ELISA for FINS and end‐point method for TC and TG. And the content of FBG, FINS, TC and TG was used for evaluating the level of HOMA‐IRI, HOMA‐β% and HOMA‐ISI. As shown in Table 2, compared with the normal group, levels of FBG, FINS, TC, TG, glyeosylated haemoglobin (HbAlc) and HOMA‐IR were observed to be increased in other six groups, while the trends of HOMA‐β% and HOMA‐ISI were on the opposite. Compared with the GDM and NC groups, levels of FBG, FINS, TC, TG, HbAlc and HOMA‐IR declined, and HOMA‐β% and HOMA‐ISI raised in the miR‐351 mimic, siRNA‐FLOT2 and miR‐351 inhibitor + siRNA‐FLOT2 groups (all *P* < 0.05), while an opposite trend was found in the miR‐351 inhibitor group (all *P* < 0.05). In contrast to the siRNA‐FLOT2 group, levels of FBG, FINS, TC, TG, HbAlc and HOMA‐IR increased, and levels of HOMA‐β% and HOMA‐ISI declined in the miR‐351 inhibitor + siRNA‐FLOT2 group (all *P* < 0.05). From these results, it was suggested that the insulin sensitivity and islet β‐cell function could be promoted by overexpression of miR‐351 and silencing of FLOT2.

**Table 2:** MiR‐351 overexpression and FLOT2 silencing promote insulin sensitivity and rescued islet β‐cell function in GDM mice

Biochemical index	Normal	Blank	NC	miR‐351 mimic	miR‐351 inhibitor	siRNA‐FLOT2	miR‐351 inhibitor +siRNA‐FLOT2
FBG (mmol/L)	4.02 ± 0.05	5.38 ± 0.15^a^	5.34 ± 0.16^a^	4.42 ± 0.12^a^, ^b^	6.26 ± 0.13^a^, ^b^	4.45 ± 0.11^a^, ^b^	4.86 ± 0.12^a^, ^b^
FINS (μU/mL)	10.05 ± 0.08	21.14 ± 0.73^a^	20.98 ± 0.67^a^	14.67 ± 0.22^a^, ^b^	23.32 ± 0.85^a^, ^b^	14.70 ± 0.24^a^, ^b^	18.25 ± 0.24^a^, ^b^, ^c^
TC (OD value)	2.25 ± 0.07	3.52 ± 0.18^a^	3.47 ± 0.17^a^	2.79 ± 0.12^a^, ^b^	4.65 ± 0.28^a^, ^b^	2.82 ± 0.15^a^, ^b^	3.21 ± 0.13^a^, ^b^, ^c^
TG (OD value)	0.92 ± 0.14	2.58 ± 0.25^a^	2.7 ± 0.26^a^	1.46 ± 0.18^a^, ^b^	3.12 ± 0.31^a^, ^b^	1.51 ± 0.19^a^, ^b^	2.03 ± 0.21^a^, ^b^, ^c^
HbAlc (%)	1.58 ± 0.33	5.97 ± 0.62^a^	5.64 ± 0.38^a^	3.77 ± 0.45^a^, ^b^	7.12 ± 0.92^a^, ^b^	3.52 ± 0.43^a^, ^b^	4.88 ± 0.30^a^, ^b^, ^c^
HOMA‐IR	1.80 ± 0.02	5.05 ± 0.18^a^	4.98 ± 0.26^a^	2.88 ± 0.07^a^, ^b^	6.49 ± 0.33^a^, ^b^	2.91 ± 0.07^a^, ^b^	3.94 ± 0.13^a^, ^b^, ^c^
HOMA‐β%	389.71 ± 39.14	226.18 ± 21.04^a^	229.21 ± 18.14^a^	323.74 ± 41.95^a^, ^b^	169.17 ± 6.95^a^, ^b^	313.26 ± 39.95^a^, ^b^	270.43 ± 3.22^a^, ^b^, ^c^
HOMA‐ISI	−3.70 ± 0.01	−4.73 ± 0.04^a^	−4.72 ± 0.05^a^	−4.17 ± 0.02^a^, ^b^	−4.98 ± 0.05^a^, ^b^	−4.18 ± 0.02^a^, ^b^	−4.48 ± 0.03^a^, ^b^, ^c^

Notes

One‐way anova was performed for statistical analysis, and the experiment was repeated three times (n = 10).

FBG, fasting blood glucose; FINS, fasting insulin; FLOT2, flotillin 2; GDM, gestational diabetes mellitus; HbAlc, glyeosylated haemoglobin; HOMA‐β%, HOMA for islet β‐cell function; HOMA‐IR, homeostasis model of assessment for insulin resistance index; HOMA‐ISI, HOMA for insulin sensitivity index; miR‐351, microRNA‐351; NC, negative control; OD, optical density; siRNA, scramble small interfering RNA; TC, total cholesterol; TG, triglyceride.

a *P* < 0.05 vs the normal group.

b *P* < 0.05 vs the GDM and NC groups.

c *P* < 0.05 vs the siRNA‐FLOT2 group.

### 3.6 MiR‐351 inhibits the PI3K/AKT pathway by down‐regulating FLOT2 in GDM mice

**Figure 5:**
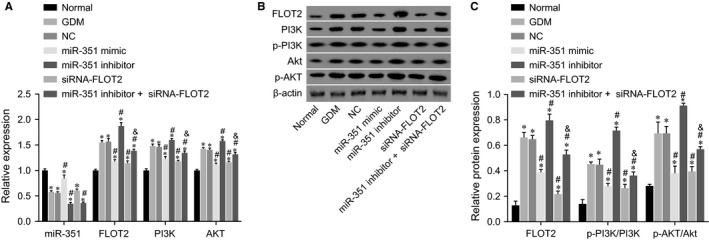
Overexpression of miR‐351 inhibits the PI3K/AKT pathway by down‐regulating FLOT2. (A) miR‐351 expression and mRNA levels of FLOT2, PI3K and AKT in response to the treatment of miR‐351 mimic, miR‐351 inhibitor, siRNA‐FLOT2, miR‐351 inhibitor + siRNA‐FLOT2; (B) Western blot bands of FLOT2, PI3K, AKT, p‐PI3K, p‐AKT and β‐actin protein in response to the treatment of miR‐351 mimic, miR‐351 inhibitor, siRNA‐FLOT2, miR‐351 inhibitor + siRNA‐FLOT2; C, protein levels of FLOT2, the extent of PI3K and AKT phosphorylation in response to the treatment of miR‐351 mimic, miR‐351 inhibitor, siRNA‐FLOT2, miR‐351 inhibitor + siRNA‐FLOT2; *, *P* < 0.05 vs the normal group; #, *P* < 0.05 vs the GDM and NC groups; ^&^, *P* < 0.05 vs the siRNA‐FLOT2 group; RT‐qPCR, reverse transcription quantitative polymerase chain reaction; miR‐351, microRNA‐351; FLOT2, flotillin 2; PI3K, phosphoinositide 3‐kinase; AKT, protein kinase B; p‐PI3K, PI3K phosphorylation; p‐AKT, AKT phosphorylation; NC, negative control; GDM, gestational diabetes mellitus; siRNA, small interfering RNA; anova, analysis of variance. The one‐way anova was performed for statistical analysis, and the experiment was repeated three times (n = 10)

Reverse transcription quantitative polymerase chain reaction (RT‐qPCR) and Western blot analysis were applied to determine the levels of PI3K, AKT and FLOT2 as well as the extent of PI3K and AKT phosphorylation after different transfection. As shown in Figure 5 A‐C, the levels of PI3K, AKT and FLOT2 as well as the extent of PI3K and AKT phosphorylation were elevated, while miR‐351 expression was decreased in other six groups when compared with the normal group (all *P* < 0.05). In contrast to the GDM and NC groups, the miR‐351 mimic, siRNA‐FLOT2 and miR‐351 inhibitor + siRNA‐FLOT2 groups showed reduced levels of PI3K, AKT and FLOT2 together with decreased extent of PI3K and AKT phosphorylation (all *P* < 0.05), while an opposite trend was revealed in the miR‐351 inhibitor group (*P* < 0.05). The expression of miR‐351 increased in the miR‐351 mimic group while decreased in the miR‐351 inhibitor + siRNA‐FLOT2 group (all *P* < 0.05), and no significant difference was found in the siRNA‐FLOT2 group (*P* > 0.05). In comparison with the siRNA‐FLOT2 group, the levels of PI3K, AKT and FLOT2 as well as the extent of PI3K and AKT phosphorylation increased in the miR‐351 inhibitor +siRNA‐FLOT2 group (all *P* < 0.05). Another promising finding was that inhibition of the PI3K/AKT pathway could be triggered by miR‐351 overexpression targeting FLOT2 in GDM mice.

### 3.7 MiR‐351 up‐regulation and FLOT2 silencing alleviate IR and gluconeogenesis in liver of GDM mice

**Figure 6:**
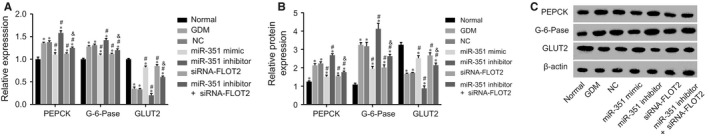
IR and gluconeogenesis is suppressed by miR‐351 overexpression and FLOT2 silencing. (A) mRNA levels of PEPCK, G‐6‐Pase and GLUT2 in response to the treatment of miR‐351 mimic, miR‐351 inhibitor, siRNA‐FLOT2, miR‐351 inhibitor + siRNA‐FLOT2; (B) protein levels of PEPCK, G‐6‐Pase and GLUT2 in response to the treatment of miR‐351 mimic, miR‐351 inhibitor, siRNA‐FLOT2, miR‐351 inhibitor + siRNA‐FLOT2; (C) grey value of PEPCK, G‐6‐Pase, GLUT2 and β‐actin protein bands in response to the treatment of miR‐351 mimic, miR‐351 inhibitor, siRNA‐FLOT2, miR‐351 inhibitor + siRNA‐FLOT2. *, *P* < 0.05 vs the normal group; #, *P* < 0.05 vs the GDM and NC groups; &, *P* < 0.05 vs the siRNA‐FLOT2 group; IR, insulin resistance; RT‐qPCR, reverse transcription quantitative polymerase chain reaction; miR‐351, microRNA‐351; FLOT2, flotillin 2; PEPCK, phosphoenolpyruvate carboxykinase; G‐6‐Pase, glucose‐6‐phosphatase; GLUT2, glucose transporter 2; GDM, gestational diabetes mellitus; IR, insulin resistance; siRNA, small interfering RNA; NC, negative control. The one‐way anova was performed for statistical analysis, and the experiment was repeated three times (n = 10)

Reverse transcription quantitative polymerase chain reaction and Western blot analysis were adopted to evaluate the mRNA and protein levels of PEPCK, G‐6‐Pase and GLUT2 after different transfection. As shown in Figure 6A‐C, the levels of PEPCK and G‐6‐Pase increased, and level of GLUT2 was decreased in other six groups when compared with the normal group (all *P* < 0.05). In contrast to the GDM and NC groups, the miR‐351 mimic, siRNA‐FLOT2 and miR‐351 inhibitor + siRNA‐FLOT2 groups showed decreased mRNA and protein levels of PEPCK and G‐6‐Pase together with increased mRNA and protein level of GLUT2 (all *P* < 0.05), while an opposite trend was revealed in the miR‐351 inhibitor group (*P* < 0.05). The mRNA and protein levels of FLOT2, PEPCK and G‐6‐Pase increased, while that of GLUT2 decreased in the miR‐351 inhibitor + siRNA‐FLOT2 in comparison with the siRNA‐FLOT2 group (*P* < 0.05). Together, the present findings confirmed that FLOT2 down‐regulation resulted by miR‐351 overexpression could contribute to alleviation of IR and suppression of gluconeogenesis in liver of GDM mice according to the decrement of PEPCK and G‐6‐Pase and increment of GLUT2.

## 4 DISCUSSION

Gestational diabetes mellitus is known as a severity of glucose intolerance which is initially diagnosed during pregnancy, and it is increasingly prevalent among Chinese female population as a pivotal public health problem.^32^, ^33^ Significantly, GDM without recognition and treatment is likely to bring maternal and foetal consequences, so it needs to be early detected and treated.^34^ Treatment to reduce blood glucose concentration alone or together with special obstetric care seems to effectively avoid the possibility of some perinatal complications, thus ameliorating GDM.^35^ Nevertheless, more efficient way of treatment is required. At current time, we investigate the possible involvement of miR‐351 in GDM by interacting with FLOT2 through PI3K/AKT pathway, which may provide useful functional insights. After our experiments were carried out, these findings demonstrate that high expression of miR‐351 could negatively regulate PI3K/AKT pathway interacting with FLOT2, thereby acting as an inhibitor of GDM.

The initial discovery in our study is that miR‐351 is poorly expressed while FLOT2 is highly expressed in GDM. Several earlier studies implied that miR‐146a, miR‐20b, miR‐21, miR‐24, miR‐15a, miR‐126, miR‐191, miR‐197, miR‐223, miR‐320 and miR‐486 levels were seen a reduction in prevalent diabetes.^36^, ^37^ Apart from that, miR‐21a and miR‐93 levels are observed to be decreased in peripheral blood mononuclear cells in patients diagnosed with type 1 diabetes.^38^ It is noted that miR‐351 is a family member of the INFβ‐inducible mRNAs, and is able to accelerate cellular activities related to antivirus and muscle regeneration.^19^ Results of prediction from bioinformatics website confirmed that FLOT2 was in targeting relationship with miR‐351. Similarly, a former paper implicated that in the invasion and migration of lung adenocarcinoma, miR‐133 targeted with FLOT2.^39^ Flotillins, also named as reggie proteins, consist of FLOT1 and FLOT2, two commonly expressed and highly conserved proteins.^40^ FLOT2, initially found to occur in goldfish, is commonly expressed in tissues of mammals and encodes a membrane protein correlated with caveolae.^38^ FLOT2 is shown to be regulated by miR‐802 and miR‐449a.^41^, ^42^ Besides, a prior study revealed that FLOT2 was an insulin pathway in T2DM.^13^ More importantly, FLOT2 has been proved as a factor related with β cell function, while IR and β cell function are known as two important pathological factors for diabetes mellitus occurrence.^43^ Furthermore, GSE87295 database in our study revealed that FLOT2 was up‐regulated in GDM patients compared with the normal people.

Furthermore, our study provided evidence that miR‐351 overexpression inhibits the PI3K/AKT pathway by suppressing FLOT2. As an inhibitor in a glucose uptake pathway,^44^ the AKT pathway has been reported to regulate the invasion and migration of lung adenocarcinoma cells by targeting FLOT2.^39^ In meantime, FLOT2 exerts a cancerous role through the PI3K/AKT3 pathway, thus accelerating the nasopharyngeal carcinoma cell cycle.^14^ Similar to our finding, miR‐485 exerts a negative function on metastasis and epithelial‐mesenchymal transition in lung adenocarcinoma by targeting FLOT2 via the PI3K/AKT pathway.^45^ Epidermal growth factor‐regulated activation of the PI3K/AKT pathway has been verified to be of great importance in promoting placenta development and foetal growth in humans and rodents.^29^ PI3K/AKT pathway activation reverses IR and promotes glycometabolism by a novel formula Sang‐Tong‐Jian (STJ) in T2DM.^46^ Liuwei Dihuang decoction, a traditional Chinese medicine formula, intervenes IR by down‐regulating the PI3K/AKT pathway of T2DM rats in liver.^47^ Moreover, PI3K/AKT signalling has been proved to play a role in cardiomyocyte apoptosis induced by high glucose.^48^ In this study, insulin stimulated PI3K/AKT pathway. Then diabetes PI3K/AKT pathway was activated in GDM (due to increased insulin level) yet did not translate to improved glycemic effect, suggesting the incidence of IR. These findings came to a demonstration that miR‐351 negatively regulated PI3K/AKT pathway by down‐regulating FLOT2. Another intriguing finding emerging in this study is that miR‐351 overexpression attenuates IR and gluconeogenesis in liver according to the decrease of PEPCK and G‐6‐Pase and increase of GLUT2. The close relationship between PEPCK and IR has been well investigated and identified.^49^ PEPCK exerts a role in development of IR in mice and confers high susceptibility to det‐induced IR,^50^, ^51^ which is identified to be a significant indicator for diagnosing and treating diabetes regulated by the PI3K/AKT pathway.^52^ Both PEPCK and G‐6‐Pase are in tight and positive association with gluconeogenesis.^53^, ^54^ Similar to our finding, miR‐877‐5p is inversely correlated with PEPCK expression and miR‐451 negatively modulated G‐6‐Pase expression.^55^, ^56^ The primary hepatic liver transporter GLUT2 is a transporter with low affinity and high capacity which is closely related to insulin receptor.^57^ AKT, a significant downstream node of the PI3K/AKT pathway is responsible for altering PEPCK and G‐6‐Pase expression levels via Forkhead box protein O1.^58^ Besides, the PI3K/AKT pathway is also implicated in up‐regulation of the expression of GLUT2 and GLUT4 via STJ.^46^ Taken together, we can make a conclusion here that miR‐351 overexpression decreases PEPCK and G‐6‐Pase, and increases GLUT2, thus improving insulin sensitivity and inhibiting gluconeogenesis in liver.

To be concluded, these preliminary results found that miR‐351 eases IR and liver gluconeogenesis in GDM via PI3K/AKT pathway by down‐regulating FLOT2 (Figure S1). With the aim to overcome some of the limitations of our study, further work needs to be done on larger samples and on treatment of patients with GDM. Studies focusing on mechanisms of action underlying would better characterize the role of miR‐351 targeted FLOT2 via PI3K/AKT pathway.

## CONFLICT OF INTEREST

None.
