# Supplementary material for: MicroRNA‐351 eases insulin resistance and liver gluconeogenesis via the PI3K/AKT pathway by inhibiting FLOT2 in mice of gestational diabetes mellitus
Source: J Cell Mol Med. 2019 Jul 9;23(9):5895–906. doi: 10.1111/jcmm.14079 (PMC6714143; doi:10.1111/jcmm.14079)
Supplement: Supplementary file 2 [file JCMM-23-5895-s002.docx]

**SUPPLEMENTARY FIGURE 1** MiR-351 targets FLOT2 in IR and liver gluconeogenesis in GDM. Our study demonstrated that the overexpression of miR-351 inhibited the expression of FLOT2, thus downregulating the related genes of the PI3K/AKT pathway (including PI3K and AKT), thereby mediating the expression of IR- and liver gluconeogenesis-related genes (including PEPCK, G-6-Pase and GLUT2) and ultimately impeding IR and liver gluconeogenesis in GDM. MiR-351, microRNA-351; PI3K, phosphoinositide 3-kinase; AKT, protein kinase; FLOT2, flotillin 2; GDM, gestational diabetes mellitus; PEPCK, phosphoenolpyruvate carboxykinase; G-6-Pase, glucose-6-phosphatase; GLUT2, glucose transporter 2; IR, insulin resistance.
